# Climate change stimulates the growth of the intertidal macroalgae *Ascophyllum nodosum* near the northern distribution limit

**DOI:** 10.1007/s13280-016-0873-7

**Published:** 2017-01-23

**Authors:** Núria Marbà, Dorte Krause-Jensen, Birgit Olesen, Peter B. Christensen, Anissa Merzouk, Joao Rodrigues, Susse Wegeberg, Robert T. Wilce

**Affiliations:** 1 Department of Global Change Research, IMEDEA (CSIC-UIB), Miquel Marquès 21, 07190 Esporles (Illes Balears), Spain; 2 Department of Bioscience, Aarhus University, Vejlsøvej 25, 8600 Silkeborg, Denmark; 3 Arctic Research Centre, Aarhus University, Ny Munkegade 114, Building 1540, 8000 Århus C, Denmark; 4 Department of Bioscience, Aarhus University, Ole Worms Allé 1, Building 1135, 8000 Aarhus C, Denmark; 5 ArcticNet/Amundsen Science, Université Laval, Pavillon Alexandre-Vachon, Room 4081, 1045 Avenue de la Médecine, Quebec, QC G1V 0A6 Canada; 6 St Catharine’s College, University of Cambridge, Cambridge, CB2 1RL UK; 7 Department of Bioscience, Aarhus University, Frederiksborgvej 399, 4000 Roskilde, Denmark; 8 Department of Biology, University of Massachusetts, Amherst, MA 01003 USA

**Keywords:** Elongation, Greenland, Latitude gradient, Norway, Warming

## Abstract

*Ascophyllum nodosum* is a foundation macroalgae of the intertidal zone that distributes across latitude 41.3–69.7°N. We tested the hypothesis that growth of *A. nodosum* near the northern distribution edge increases with warming. We retrospectively quantified the growth of eight *A. nodosum* populations at West Greenland and North Norway (from 64°N to 69°N). For seven populations, we measured growth rates since 1997–2002 and for one of them we extended the time series back to 1956 using published estimates. Individuals at northern populations elongated between 2.0 and 9.1 cm year^−1^ and this variability correlated with temperature and annual ice-free days. A spatial comparison of *A. nodosum* growth across the species distribution range showed that Northern (and coldest) populations grew at the slowest rates. Our results demonstrate that arctic climate change enhances the growth of *A. nodosum* populations and suggest that their productivity may increase in response to projected global warming.

**Electronic supplementary material:**

The online version of this article (doi:10.1007/s13280-016-0873-7) contains supplementary material, which is available to authorized users.

## Introduction

The arctic climate is rapidly changing due to excessive anthropogenic greenhouse gas emissions to the atmosphere (IPCC 2014). The Arctic Ocean ranks amongst the oceans warming at the fastest rate, twice as fast as the global ocean, and it experiences the largest thermal marine seasonal shifts recorded globally since 1960 (Burrows et al. 2011). Arctic warming is also evidenced by the rapid loss of sea ice cover during the last decades, which reached the minimum extension ever recorded in 2012 (Vaughan et al. 2013). Arctic warming is expected to continue and, even under a mild scenario of greenhouse gas emissions (i.e., IPCC scenario RCP4.5), global models project a median of 4.9 °C (maximum 9.3 °C) warming by the end of the twenty-first Century (Christensen et al. 2013).

Footprints of current arctic climate change are already evident in marine arctic ecosystems (Wassmann et al. 2011). Yet, climate change impacts on arctic macroalgal communities remain largely unexplored, despite knowledge about macroalgal responses to climate change being particularly relevant for forecasting the future functioning of coastal arctic ecosystems (Krause-Jensen and Duarte 2014). A major limiting factor is the sparsity of long-term datasets on arctic benthic vegetation, which are limited to scattered information from Svalbard fjords (Weslawski et al. 2010; Fredriksen and Kile 2012; Fredriksen et al. 2014; Kortsch et al. 2012; Bartsch et al. 2016), Greenland coasts (Krause-Jensen et al. 2012; Olesen et al. 2015) and Canadian coasts (Merzouk and Johnson 2011).

Macroalgae, mostly phaeophyta, are the dominant marine vegetation along the arctic and sub-arctic coasts, where they form lush and productive populations (Pedersen 2011; Krause-Jensen et al. 2012) and provide important ecological goods and services such as primary production, nutrient retention, habitat complexity, marine biodiversity, fertiliser and food production (Steneck et al. 2002; Schmidt et al. 2011). Macroalgal growth, survival and reproduction in northern regions are largely controlled by climatic conditions such as light, temperature and, for intertidal species, also icebergs and sea ice scour (e.g., Zacher et al. 2009; Wiencke and Amsler 2012). Climate conditions along the sub-arctic and arctic coasts reach extreme values and exhibit wide seasonal fluctuations partly due to the arctic dark season in combination with the development of sea ice that result into days to months of 24 h darkness above the Arctic Circle (66°N). Ice scour from icebergs and pack ice particularly impacts intertidal rocky shores, and, thus, lush intertidal seaweed populations mostly develop in sheltered areas. Latitudinal variations in climatic conditions along the sub-arctic and arctic regions determine the position of biogeographical distribution edges of species (Müller et al. 2009) and may constrain macroalgal growth and productivity as observed in kelp forests (Krause-Jensen et al. 2012). Recent studies document poleward migration of geographical distribution ranges of marine biota, including macroalgae, in response to ocean warming (Poloczanska et al. 2013; Yesson et al. 2015; Straub et al. 2016). There is limited in situ documentation of responses of macroalgae along sub-arctic and arctic coasts. Instead, laboratory studies of temperature tolerance and response are used as a basis for predictions of future distribution limits and response to climate change (e.g., Müller et al. 2009; Wilson et al. 2015; Wilce 2016).

The brown macroalga *Ascophyllum nodosum* is a key foundation species, as it plays a strong role in structuring coastal communities (Schmidt et al. 2011), that occurs along sheltered intertidal rocky shores of North Atlantic coasts, from 41.3°N to 69.7°N (South and Titley 1986; Lüning 1990). Greenland, North Norway and South Baffin Island (Canada) host the northernmost *A. nodosum* populations described to date (Lüning 1990; Pedersen 2011). The broad geographical distribution of *A. nodosum* reflects its wide thermal tolerance, which ranges from less than 0 °C and up to about 25 °C with optimal temperature at around 15 °C (Fortes and Lüning 1980), considerably above current temperatures in the sub-arctic and arctic region. *A. nodosum* is a branched perennial species, with thalli that persist for 10–20 years (Stengel and Dring 1997) and the lifespan of individuals modelled at up to 300 years (Åberg 1992). Vegetative growth occurs primarily through elongation of the tips of the thallus while producing annually one bladder per tip (Macfarlane 1933). The growth form and architecture of *A. nodosum* thereby enable to retrospectively quantify growth of the thallus based on a single sampling event, and explore the possible drivers (e.g., climate change) of decadal variability in the growth records. Hence, the retrospective assessment of *A. nodosum* growth in relation to temperature changes can help assessing the possible impact of arctic climate change on its populations, even at remote sites such as the northern edge of the distribution range. This approach as has been done in the past for the arctic cockle *Clinocardium ciliatum* to test the effect of the length of sea ice season on annual growth (Sejr et al. 2009). While studies of *A. nodosum* response to climate change have been conducted at the southern edge of the geographical distribution (Araújo et al. 2014; Viana et al. 2014), no similar reports exist for the northern edge of its geographical distribution. The few projections of northern range expansion do not include the northernmost Greenland *A. nodosum* populations (Jueterbock et al. 2013; Neiva et al. 2016).

Here we assess whether arctic climate (i.e., temperature, sea ice cover) change affects growth of *Ascophyllum nodosum* populations at the northern fringe of the sub-arctic. We do so by retrospectively quantifying seaweed growth in six Greenlandic and two Norwegian populations between 64.2°N and 69.2°N since 1997–2002 and examine its relation with climatic forcing. Moreover, we examine large-scale patterns in *A. nodosum* growth in relation to variability in summer seawater temperature across the entire biogeographical distribution range of the species. We discuss how projected arctic warming, under the IPCC scenarios of greenhouse gas emissions (IPCC 2014), may affect the productivity of *A. nodosum* at the northern distribution limit of the species during the twenty-first century.

## Materials and methods

### Study sites

**Fig. 1:**
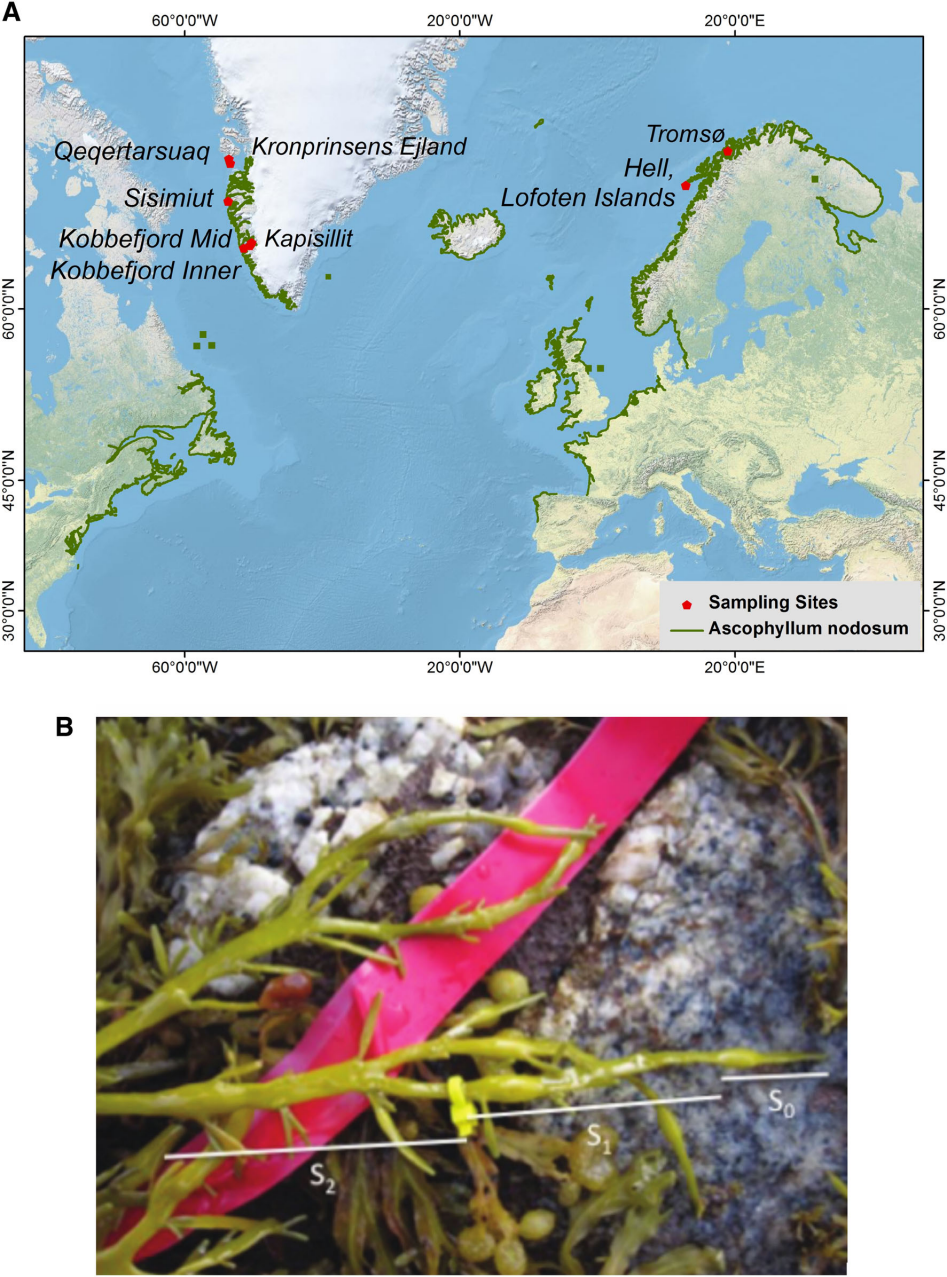
**a** Location of study sites. **b** *Ascophyllum nodosum* tip showing the three youngest internodes: *S*_0_ (produced the current year), *S*_1_ (produced the previous year), *S*_2_ (produced 2 years earlier). The *yellow marking* was used to test the assumption that a new bladder is produced every year: the production of a new bladder apparently displaced the *yellow mark* from its original the position between the youngest and the second youngest bladder to the position between the second and third youngest within 1 year

Eight sites were studied along the coasts of West Greenland and North Norway (Fig. 1a). Greenland’s west coast study sites extend from Kobbefjord (two sites) and Kapisillit in the Godthåbsfjord system, Nuuk, at 64°N, Sisimiut at 67°N to Qeqertarsuaq and Kronprinsens Ejland on/by the Disko Island at 69°N, which represent the northernmost observations of *Ascophyllum nodosum* in Greenland. We included two additional study sites in northern Norway, Hell on the Lofoten Islands (68°N) and Tromsø (69°N), i.e., at similar latitude as the northernmost Greenland sites, but subjected to the warmer waters of the Gulf Current. Our *A. nodosum* collections were made between the years 2009 and 2012 at the mid-intertidal zone in August/September except those at Tromsø which we sampled in January (Table 1).

**Table 1:** Name and coordinates of *Ascophyllum nodosum* sampling sites, name of the meteorological station nearby the sampling sites and name of the area surveyed for annual ice-free days. The dates of seaweed sampling and the starting and ending years of time series are provided. Historical information of growth was available from two sites

Site	Seaweed sampling date (day/month/year); starting and ending years of growth time series	Source annual tip elongation	Source ice-free days (site); starting and ending years of time series	Source air temperature (meteorological station); starting and ending years of time series
West Greenland				
Godthåbsfjord system				
*Kobbefjord Inner*	15/8/2010 1997–2009	1	3 (Nuuk) 1990–2009	4 (Nuuk-04250) 1958–2012
*Kobbefjord Mid*	13/8/2010 1997–2009	1	3 (Nuuk) 1990–2009	4 (Nuuk-04250) 1958–2012
*Kapisillit*	5/8/2011 2010	1	–	4 (Nuuk-04250) 1958–2012
Sisimiut	28/8/2009 2000–2008	1	3 (Sisimiut) 1990–2009	4 (Sisimiut-04230, Sisimiut-04234) 1961–2012
Disko Bay				
*Qeqertarsuaq*	29/8/2009, 1999 1997–2008	1, 2	3 (Qeqertarsuaq) 1990–2009	4 (Aasiaat-04220) 1958–2011
*Kronprinsens Ejland*	19/8/2012, 1959, 1986 1957–2011	1, 2	3 (Qeqertarsuaq) 1990–2009	4 (Aasiaat-04220) 1958–2011
Norway				
Hell, Lofoten	16/7/2010 1997–2009	1	–	5 1900–2015
Tromsø	26/1/2011 2002–2009	1	–	6 1921–2015

*1* This study, *2* Hansen et al. (2004) which also report the sampling by Wilce (1964), *3* National Snow and Ice Data Center (Cavalieri and Comiso 2004), *4* Carpenter 2013, *5* http://www.yr.no/place/Norway/Nordland/Flakstad/Lofoten/climate.html#year, *6* http://www.yr.no/place/Norway/Troms/Troms%C3%B8/Troms%C3%B8_observation_site/climate.month01.html

The dataset was further supplemented by historical observations of *A. nodosum* growth from the northernmost Greenland sites collected by R.T. Wilce at Kronprinsens Ejland in June 1959 and June 1986 and by Louise Hansen at Qeqertarsuaq in June 1999 (Hansen 2004). For the specimens collected in 1959 (*n* = 19) and 1986 (*n* = 20), growth was measured on herbarium specimens and corrected for the ca. 10% shrinking due to drying (Hansen 2004). Annual growth estimates provided by Hansen (2004) were derived from measurements on 21 individuals (Hansen 2004). Specimens collected by R.T. Wilce and L. Hansen provided growth estimates for the 2 years prior collection (i.e., years 1957, 1958, 1984, 1985 and 1997, 1998).

### Growth rates

Between 6 and 20 of the oldest thalli of *A. nodosum*, overall representing 3–17 years of growth, were harvested from all study sites except Kapisillit. On each of the collected specimens we measured the length between consecutive bladders from the tip to the base of the thallus (*S*_0_: tip to base of 1st bladder, *S*_1_: base of 1st bladder to base of 2nd bladder, *S*_2_: base of 2nd bladder to base of 3rd bladder, etc. continuing to the holdfast; Fig. 1b). These measurements retrospectively provided estimates of annual growth (assessed as tip elongation rate) of each year along the thallus lifespan. At Kapisillit, we measured the length of the youngest 3 internodes of 20–25 thalli of randomly collected individuals in the mid-intertidal.

Annual growth of *A. nodosum* was retrospectively estimated as the length of a fully grown internode, assuming that it represents 1 year of growth (MacFarlane 1933). This technique was applied to assess the growth during the year previous to collection based on the length of the youngest complete internode (*S*_1_, Fig. 1b), the growth 2 years before collection based on the length of the second youngest complete internode (*S*_2_, Fig. 1b) and so forth for the full length of the thallus. Because the youngest section of the tip (*S*_0_) did not fully grow at the time of sampling, we only included growth estimates of *S*_1_ and older segments in the analysis.

The assumption that *A. nodosum* tips produce one bladder per year was tested by marking tips at Qeqertarsuaq and Kobbefjord Mid populations. A thin cable tie was placed between the youngest and the second youngest bladder of selected thalli. One year later, the cable tie was displaced to the position between the second and third youngest bladder (Fig. 1b), hence confirming the notion that *A. nodosum* tips produce one bladder per year. The technique was further validated based on reconstructed growth estimates at Kobbefjord populations in August/September during two consecutive years (2009 and 2010). The length of the segment *S*_1_ sampled in 2009 hence matched the length of the segment *S*_2_ measured on thalli collected in 2010.

We estimated the average time series of *A. nodosum* growth for each location by calculating the mean (and standard error) of our growth measurements for each year. For some populations, we could extend the time series back in time using estimates from earlier studies at the same sites. The starting and ending year of the time series of *A. nodosum* annual growth at each location is provided in Table 1.

The dataset of *A. nodosum* annual growth was expanded with our own measurements of tip growth for 2011 at Kapisillit and annual growth estimates reported in the literature for temperate Atlantic populations. This expanded dataset was used to assess broad-scale latitudinal patterns of *A. nodosum* growth rate across the entire geographical distribution range of the species.

### Data on ice-free days and temperature

The length of the annual sea ice-free period was estimated from satellite images as described by Krause-Jensen et al. (2012) for Qeqertarsuaq, Sisimiut and Nuuk for the period 1990–2009 (Table 1). The satellite images did not allow analysis for Kapisillit, where most pixels contained overlapping land and sea information. The Norwegian sampling sites were not ice-covered. The information was obtained on the basis of sea ice concentration data obtained from passive microwave imagery processed with the Enhanced NASA Team algorithm (Markus and Cavalieri 2000), archived and distributed by the National Snow and Ice Data Center (Cavalieri and Comiso 2004). The length of the ice-free period was calculated with an algorithm described by Rodrigues (2009).

Air temperature for the Greenland sites was obtained from the Danish Meteorological Institute (DMI) (Carpenter 2013). We used DMI stations in Nuuk, Sisimiut and Aasiaat and Norwegian Meteorological Institute (MET) in Lofoten and Tromsø for coupling air temperature with *A. nodosum* data (see Table 1). DMI stations provided air temperature time series recorded every 3 h. Annual mean air temperature was calculated by averaging all measurements within each year. MET provided time series of monthly average air temperature values for the Tromsø station. Monthly means of air temperatures at Tromsø were averaged from January to December to calculate the annual mean air temperature for this station. We excluded from the time series the years without records from January to December. We used the annual mean air temperature at Lofoten station provided by MET.

### Statistical analysis

We used linear regression analysis to assess linear temporal trends in climatic variables (ice-free days, air temperature) and in *A. nodosum* growth. The regression analyses were performed on smoothed time series by a running average of 3 years. Trends in climatic variables were computed for the entire time series and for the period between 1990 and 2012, to encompass the period of *A. nodosum* growth records. Regression analysis was also used to examine the relationships between *A. nodosum* growth and climatic variables. Because the relationship between *A. nodosum* growth and ice-free days across all sites was exponential, we performed the regression analysis on ln-transformed growth estimates. The relationship between *A. nodosum* growth and latitude across the distribution range of the species was assessed with a quadratic function.

The effect of latitude (*L*) on temporal linear trends of climatic variables (mean annual air temperature or annual ice-free days) or growth variables (*C*) was tested using the model:

$$ C = a_{1} + b_{1}\cdot t + a_{2}\cdot L + b_{2} \left( {t \cdot L} \right), $$

where *a*_1_ is the intercept of the regression line of variable *C* with time (*t*) and *b*_1_ is the slope of this regression, *a*_2_ is the change in intercept when *L* is considered in the model and *b*_2_ represents the change in the slope *b*_1_ with changing *L*. JMP 10.0.0 software was used in all analyses. Statistical significance was set to *p* < 0.05.

## Results

**Fig. 2:**
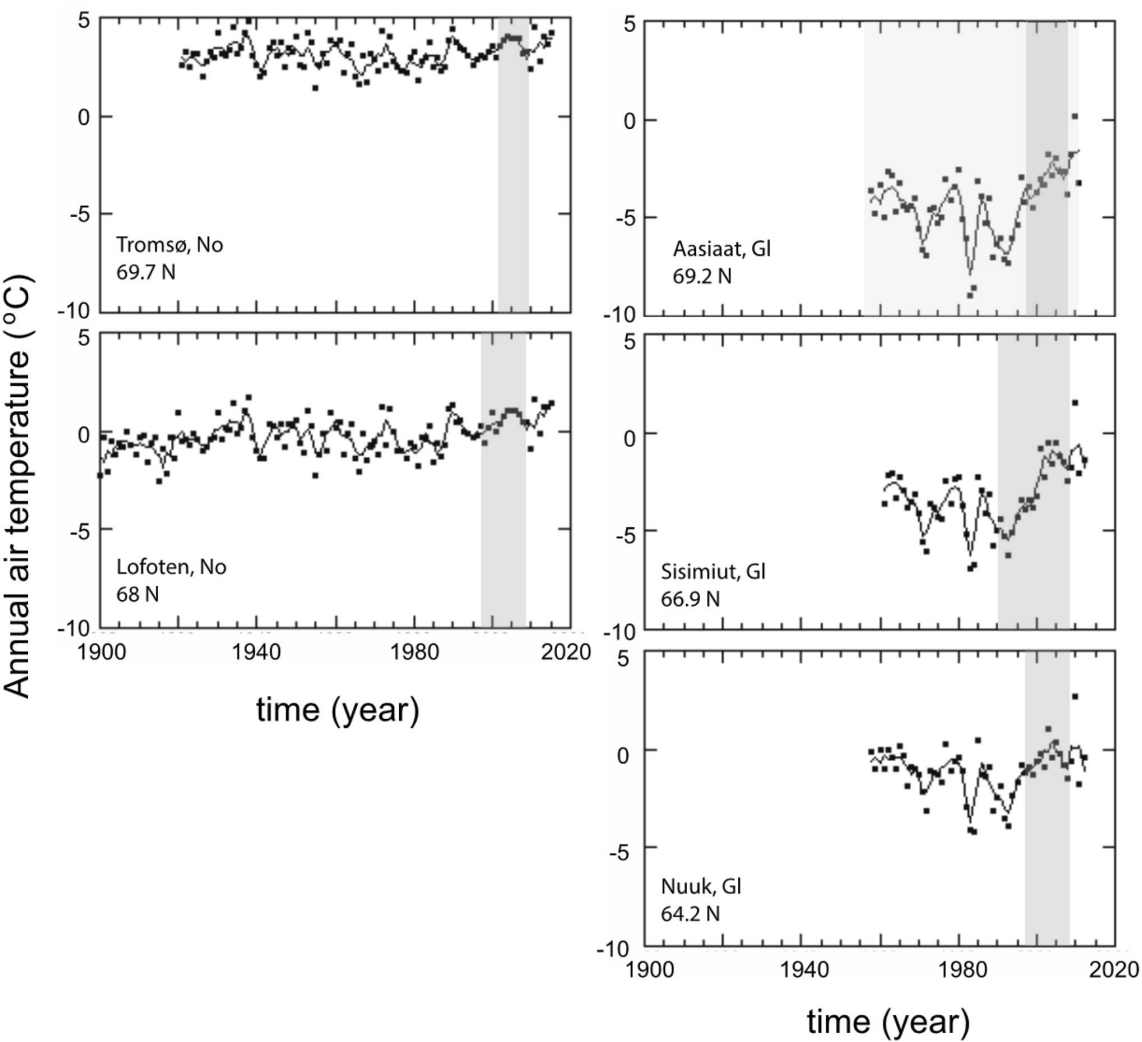
Time series of annual air temperature recorded at meteorological stations close to each study site. The dots and the solid lines show temperature observations and running mean of 3 year, respectively. Sources of data: http://www.dmi.dk/laer-om/generelt/dmi-publikationer/2013/ for Greenland (Gl), http://www.yr.no/place/Norway/Troms/Troms%C3%B8/Troms%C3%B8_observation_site/climate.month01.html for Tromsø and http://www.yr.no/place/Norway/Nordland/Flakstad/Lofoten/climate.html#year for Lofoten observations. *Grey* background indicates the time period of available *A. nodosum* growth data in the area. For Aasiaat, *A. nodosum* growth was measured at two stations and both time periods of growth measurements are indicated (Qeqertarsuaq: *dark grey*; Kronprinsens: *light grey*)

Annual mean air temperature at the studied sites since the onset of available records (Table 1) ranged between −8.9 and 4.8 °C (Fig. 2). Annual mean air temperature at each location exhibited substantial fluctuations, with the temporal pattern differing between the Norwegian and the Greenland coasts (Fig. 2). Annual mean air temperature at Lofoten, since 1900, and at Aasiaat and Sisimiut, since 1950, has significantly increased (Table 2). Warming rates ranged from 0.01 ± 0.002 °C per year (Lofoten) to 0.04 ± 0.01 °C per year (Sisimiut). Annual mean temperature after 1990 at all observatories, on average, has been between 0.23 °C (Nuuk) and 1.08 °C (Sisimiut) higher than before 1990 (Fig. 2). Since 1990, the period when most of *Ascophyllum nodosum* growth estimates are available, annual mean air temperature showed a significant monotonous increase through time in the Greenland arctic and sub-arctic sites (Table 2). At the Greenlandic sites, air warming showed a steeper increase over the last 3 decades. Significant warming rates of 0.13 ± 0.02 °C per year, at Nuuk, and more than 0.2 °C per year at Aasiaat and Sisimiut were recorded (Table S1). Warming rate did not vary across latitude (Table S2).

**Table 2:** Regression equations of significant (*p* < 0.05) temporal trends, for the entire time series and for the period since 1990 of annual mean air temperature, annual ice-free days and *Ascophyllum nodosum* annual tip elongation rate at the study sites. The equations are of the form *Y* = *a* + (*b*·*X*). The *p* value and the coefficient of determination are provided. The time periods of the series analysed are indicated. See Table S1 for regression equations with *p* value >0.05

*Y*	*X*	Site	b	SE_b_	a	SE_a_	*p* value	*R* ^2^	Time period
Mean annual air temperature (°C)	Time (year)	Lofoten	0.01	0.00	−20.44	3.21	<0.0001	0.26	1900–2015
		Aasiaat	0.03	0.01	−63.98	24.15	0.02	0.10	1953–2012
		Aasiaat	0.25	0.02	−505.86	42.63	<0.0001	0.87	1990–2012
		Sisimiut	0.04	0.01	−84.12	24.02	0.00	0.18	1961–2012
		Sisimiut	0.23	0.02	−462.28	44.03	<0.0001	0.84	1990–2012
		Nuuk	0.13	0.02	−256.08	42.40	<0.0001	0.63	1990–2012
Annual ice-free days (days year^−1^)	Time (year)	Qeqertarsuaq	9.12	1.54	−18055.37	3083.33	<0.0001	0.66	1990–2009
		Sisimiut	8.15	0.90	−16023.95	1791.25	<0.0001	0.82	1990–2009
		Nuuk	2.56	0.71	−4773.49	1422.16	0.00	0.42	1990–2009
Annual tip elongation (cm year^−1^)	Time (year)	Lofoten	0.24	0.03	−482.06	54.26	<0.0001	0.88	1997–2009
		Kronprinsens	0.09	0.02	−169.95	31.06	<0.0001	0.67	1995–2011
		Kronprinsens	0.03	0.01	−54.78	11.82	0.00	0.56	1958–2011
		Kobbe Mid	0.17	0.06	−343.55	119.77	0.01	0.44	1997–2009
Annual tip elongation (cm year^−1^)	Mean annual air temperature (°C)	Lofoten	2.02	0.50	4.12	0.30	0.00	0.60	1997–2009
		Kronprinsens	0.40	0.10	4.23	0.32	0.00	0.50	1995–2011
		Kronprinsens	0.28	0.07	3.88	0.27	0.00	0.44	1958–2011
		Qeqertarsuaq	0.44	0.18	4.28	0.55	0.04	0.41	1997–2008
		Kobbe Inner	−0.55	0.23	5.21	0.15	0.04	0.34	1997–2009
Annual tip elongation (cm year^−1^)	Annual ice-free days (days year^−1^)	Kobbe Mid	0.082	0.024	−21.84	8.57	0.01	0.51	1997–2009

**Fig. 3:**
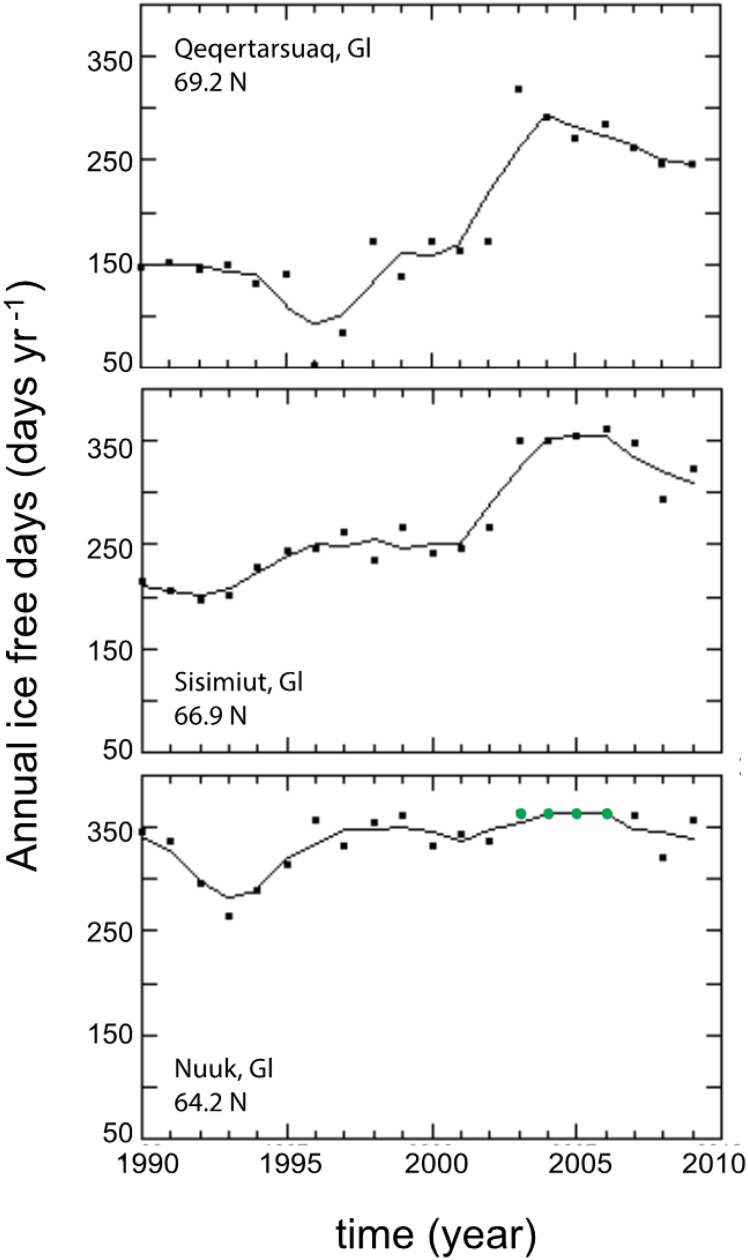
Time series of annual sea ice-free days at Greenland study sites obtained from satellite images. The dots and the *solid lines*, respectively, show the annual observations and the running mean of 3 year. *Green dots* indicate years without sea ice cover

Tromsø and Lofoten sites were free of sea ice for the entire year because the Gulf Current brings heat along the Norwegian coast. In contrast, all Greenland sites have been covered with sea ice some days of the year since 1990 (Fig. 3). The shortest annual sea ice-free period was recorded at Qeqertarsuaq and Kronprinsens Ejland (52 days). The longest annual sea ice-free period was observed at Kobbefjord sites, even though the fjord was devoid of sea ice during the period 2003–2006. Annual sea ice-free days increased at all Greenland sites since 1990 at rates varying from 2.56 ± 0.71 days per year (Kobbefjord, Nuuk) to 9.12 days per year (Qeqertarsuaq and Kronprinsens Ejland, Table 2). The rate of increase of annual sea ice-free days increased with increasing latitude (Table S2).

**Fig. 4:**
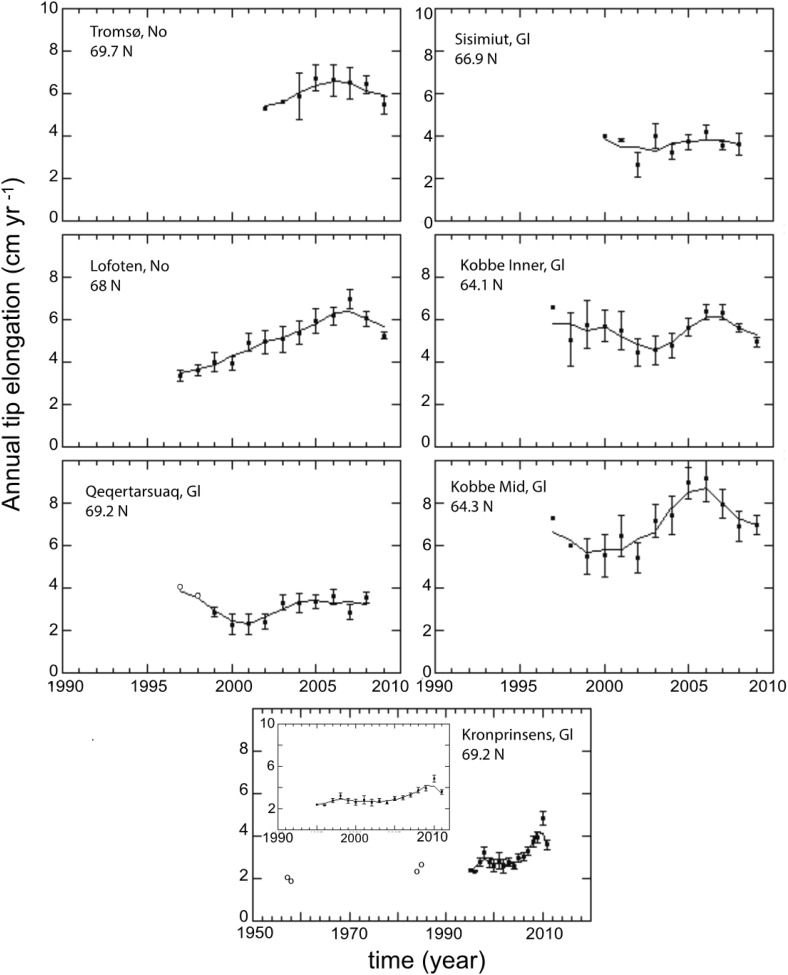
Time series of *Ascophyllum nodosum* growth at the studied sub-Arctic and Arctic populations. The *dots* and the *solid lines*, respectively, show growth observations and running mean of 3 years. Growth observations at Kronprinsens Ejland in 1957–1958 and 1984–1985 (Wilce 1964; Hansen et al. 2004) and at Qeqertarsuaq in 1997–1998 (Hansen et al. 2004) are identified by *open circles*. Growth time series at Kronprinsens Ejland since 1995 is highlighted in the inserted plot

All *A. nodosum* populations exhibited inter-annual variability of growth during the studied period. *A. nodosum* growth tended to increase towards recent time in most of the studied populations (Fig. 4). The growth rate of the tips of *A. nodosum* thalli in the arctic and sub-arctic populations for the last 1–6 decades ranged between 2.0 cm per tip and year (Kronprinsens Ejland) and 9.1 cm per tip and year (Kobbefjord Mid, Fig. 4). The fastest growth was observed after 2005 in all populations except for those at Sisimiut and Qeqertarsuaq where *A. nodosum* grew at similar rates during the period for which records are available (1997–2009, Fig. 4). *A. nodosum* growth exhibited significant temporal increasing trends in some populations. At the Kronprinsens Ejland population, that with the longest growth time series recorded, *A. nodosum* growth increased by, on average, 0.03 ± 0.01 cm every year since 1958 (Table 2). The growth rate of this population accelerated after 1990 (Table 2). A significant temporal trend of increased growth after 1990 was also observed at Lofoten and Kobbefjord Mid populations (Table 2), with the Lofoten population showing the fastest increase in annual growth (Table 2). *A. nodosum* growth tended to decrease towards the north but without a significant relationship with latitude (Table S2).

**Fig. 5:**
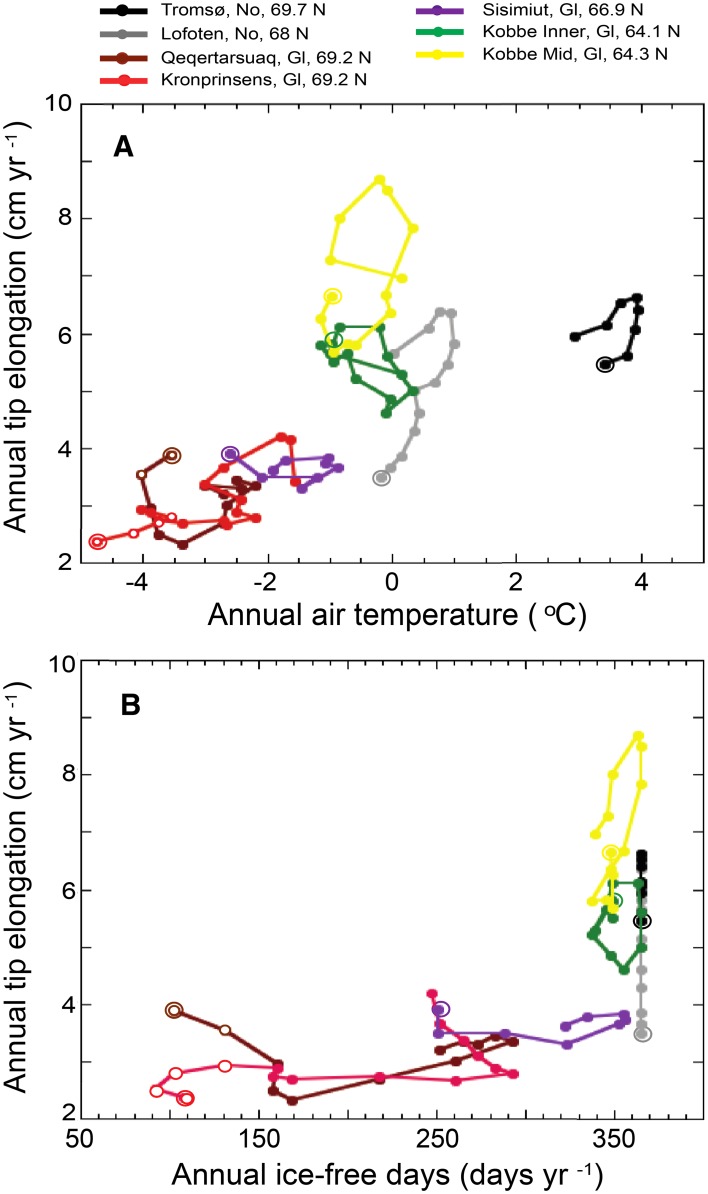
Trajectories of temporal changes in *Ascophyllum nodosum* growth with mean air temperature (**a**) and annual ice-free days (**b**). *Double circles* indicate the beginning of the time series. For all variables, the values correspond to the running mean of 3 years. *Open circles* indicate the growth estimates obtained from Wilce (1964) and Hansen et al. (2004)

Spatial and temporal variability of *A. nodosum* growth at the northern populations were coupled with annual mean air temperature. Overall annual growth of *A. nodosum* tips in these populations increased 0.47 ± 0.06 cm per degree C (regression analysis, *p* < 0.001, *R* ^2^ = 0.45, *N* = 88, data not shown) and this rate varied across populations (Table 2, Table S1). The fastest increase of *A. nodosum* annual growth per degree C of warming since 1990 was observed at the Lofoten population (Table 2, Table S1) followed by Qeqertarsuaq and Kronprinsens Ejland (Table 2, Table S1). The rate of growth increase per degree C estimated since 1990 at Kronprinsens Ejland was faster in recent years (after 1990) than for the entire period since 1958 (Table 2). Despite that *A. nodosum* annual growth and temperature were not significantly coupled at Tromsø (Table S1), the growth trajectory showed an increase with increasing air temperature and a decrease when air temperature cooled (Fig. 5a). Conversely, temporal growth changes at Sisimiut and Kobbefjord were uncoupled or negatively correlated to temperature variability (Table 2, Table S1).

Temporal and spatial variability of annual growth of *A. nodosum* correlated with that of annual sea ice-free days. Growth of *A. nodosum* increased exponentially at a rate of 0.3 ± 0.03% per day of sea ice-free cover (regression analysis, *p* < 0.001, *R* ^2^ = 0.53, *N* = 75, data not shown). Local trajectories of *A. nodosum* growth showed a clear coupling to ice-free days at Kobbefjord Mid (Table 2, Table S1) and at Qeqertarsuaq after 1997 but not at the other Greenland study sites (Fig. 5b).

**Fig. 6:**
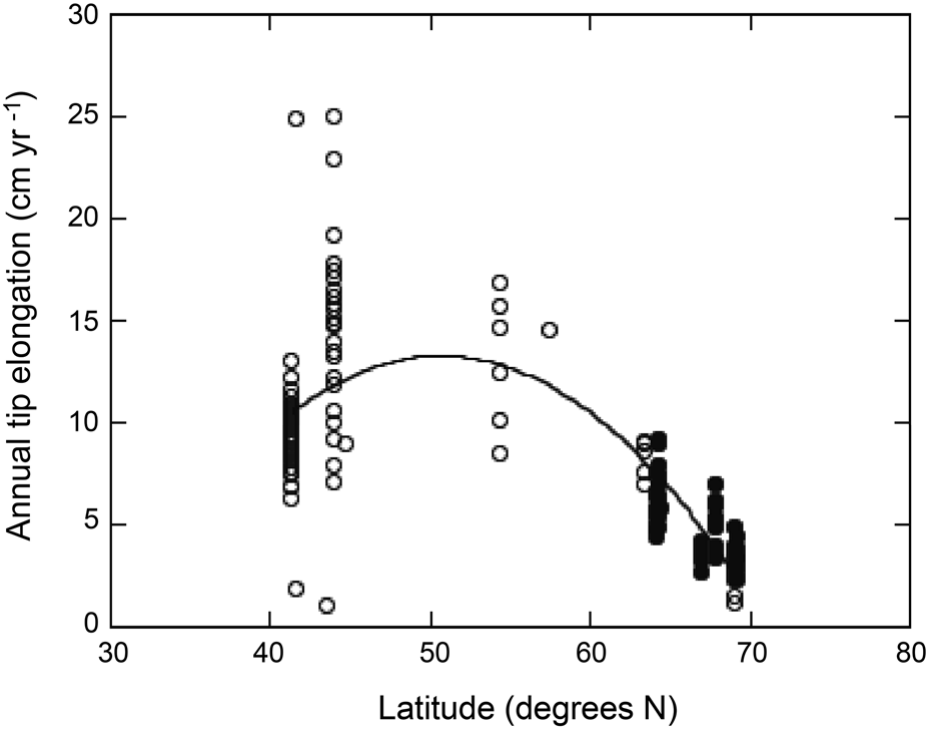
*Ascophyllum nodosum* annual tip elongation versus latitude. *Filled circles* indicate the annual elongation rate of seaweed tips of the studied populations. The *solid line* indicates the equation fitted *y* = 30.42 (±1.47) − [0.32 (±0.02)·*x*] − [0.03 (±0.004)·(*x* − 55.72)^2^] (*n* = 172, *R* ^2^ = 0.61, *p* < 0.0001)

*A. nodosum* populations located at the northern edge of the species occurrence grew 2.4 times slower than populations located further south, i.e., along the Atlantic coasts (Fig. 6). Indeed, annual growth rate of *A. nodosum* tended to increase from populations at 40^o^N to those growing at 55^o^N, where maximum growth rates were reported (Fig. 6a). Above 60^o^N, *A. nodosum* growth rate rapidly decreased the further north the populations were located (Fig. 6).

## Discussion

This study reports the longest time series on seaweed growth in the sub-arctic region so far published, spanning from one to six decades at the northern edge of *Ascophyllum nodosum*’s distribution range. The longest time series documented significant increases in *A. nodosum* growth rates over time with the fastest increases occurring since 1990. The temporal trends of faster growth of *A. nodosum* were in agreement with general trends of warming and longer ice-free periods (at sites with seasonal ice cover). All the northern populations above 68°N (i.e., Qeqertarsuaq, Kronprinsens Ejland, Lofoten; except Tromsø for which we have only had a short time series) showed a significantly positive temporal increase in annual growth with air warming (Table 2). We also saw a latitudinal trend of increased growth of sub-arctic *A. nodosum* populations from north to south along Greenland’s coast matching the higher temperatures and the reduced sea ice cover towards the south. In addition, our data clearly show that *A. nodosum* populations grow much faster at 69°N along Norway’s Gulf Current-influenced coast, than at the same latitude in the colder and partly ice-covered Greenland waters.

Our results show that substantial variability in *A. nodosum* growth at northern populations correlates with temperature (45%) and ice-free days (53%) variability. However, these results also indicate that other environmental factors cause variability in growth. For instance, salinity, which constrains the distribution of macroalgae (Nielsen et al. 1995), varies from 28 to 33‰ in Kobbefjord Mid during the growing season (spring–summer; Sørensen et al. 2015) and may explain the relatively slow growth rates at this site. The growth season of populations located at Lofoten and Tromsø (Norway) is shorter than that at Kobbefjord because of their higher latitudinal location, and this could limit their annual growth rate, despite being exposed to higher temperatures. The degree of wave exposure could also constrain intertidal macroalgal growth since it has been demonstrated that wave exposure increases the stress of rocky shore intertidal communities (Scrosati and Heaven 2007).

Our *A. nodosum* results are in accord with trends reported for other sub-arctic littoral macroalgal communities in response to arctic warming (Weslawski et al. 2010; Fredriksen and Kile 2012; Fredriksen et al. 2014). Our results also agree with reported trends for sub-arctic subtidal macroalgal communities of increased cover and diversity in Svalbard fjords from 1980 to 2003 (Beuchel et al. 2006) and from 1980 to 2010 (Kortsch et al. 2012), increased macroalgal biomass in other Svalbard fjords over the period 1996–2013 (Bartsch et al. 2016) and increased growth of kelp in response to reduced sea ice cover in North East Greenland over the period 1999–2011 (Krause-Jensen et al. 2012). Some of these studies point at intertidal macroalgal communities being more responsive to climate changes than sublittoral communities. Indeed, Helmuth et al. (2006) suggested that intertidal communities are particularly sensitive to changing temperatures.

These results suggest that *A. nodosum* communities are excellent bioindicators of climate change in the marine arctic, and that the estimation of tip growth of *A. nodosum* may be used as a relevant parameter in sub-arctic and arctic marine monitoring programmes. In fact, the Greenland Ecosystem Monitoring Programme^1^ has already adopted *A. nodosum* tip growth as an important marine bioindicator of environmental change in the Nuuk area, West Greenland.

*A. nodosum* populations at the northern fringe of sub-arctic are growing at the slowest rates when compared with those of the species across its the biogeographical distribution range (Fig. 6), which further underlines the stimulating role of temperature. Seawater summer temperature at the northern *A. nodosum* population ranges from 6 to 14 °C whereas *A. nodosum* populations below 50^o^N, exhibiting maximum growth rates, are exposed to seawater summer temperature exceeding 21 °C (Keser and Larson 1984; Peckol et al. 1988; Keser et al. 2005). Independent studies support the positive effect of warming on *A. nodosum* growth until an upper threshold is reached (Fortes and Lüning 1980; Keser et al. 2005; Wilson et al. 2015). An assessment of changes in the abundance of large brown seaweeds across the British Isles over past four decades reports overall favourable effects of warmer summer and winter temperatures on *A. nodosum* where mean summer temperatures reach a maximum of about 16 °C (Yesson et al. 2015). Long-term (1979–2002) in situ studies of *A. nodosum* growth in Connecticut, USA, also reported enhanced seaweed growth with warming until temperatures reached 25 °C. Growth rates decreased rapidly and mortality increased as temperatures exceeded 27–28 °C (Keser et al. 2005). These findings were confirmed experimentally, when warming above 23 °C resulted in reduced growth and simulations of heat waves (26, 29 °C) increased the mortality of *A. nodosum* in Nova Scotia, Atlantic Canada (Wilson et al. 2015). Both studies are in line with the fact that we found no studies of in situ *A. nodosum* growth at summer temperatures above 24 °C. Longphuirt et al. (2013) further reported that *A. nodosum* exhibits higher CO_2_ affinity at higher temperature, suggesting a seasonal strategy of photosynthetic up-regulation during the growth period. Competitive interactions amongst coexisting intertidal species may also change with warming and affect *A. nodosum* distribution patterns. Indeed, the increased occurrence of *Fucus vesiculosus* in *A. nodosum* beds has been attributed to synergetic effect of sea surface warming combined with harvesting (Ugarte et al. 2010).

Recent studies also suggest some poleward migration of *A. nodosum* in response to warming based on knowledge on temperature tolerance in combination with predicted future isotherm migration (Jueterbock et al. 2013; Neiva et al. 2016). However, these studies do not consider the northernmost Greenland populations neither in the current distribution maps nor in future scenarios. Also the scenarios for *Ascophyllum* distribution in year 2100 and 2200 suggest retreat of some populations even along the northern distribution limit (Jueterbock et al. 2013; Neiva et al. 2016). Our results suggest that warming of coastal water would lead to an overall stimulation and expansion of the northernmost populations of *A. nodosum*. Future distribution boundaries and the speed of northward migration will, however, also depend heavily on the dispersal capacity of the species.

## Conclusion

Our results demonstrate a marked positive response of growth rates of *A. nodosum* populations to warming at the northern fringe of the sub-arctic. *A. nodosum* as well as the majority of other key species of marine vegetation in Greenland are cold-temperate species with optimum temperatures considerably above current temperature regimes of cold boreal coastal waters (Fortes and Lüning 1980; Müller et al. 2009; Wiencke and Amsler 2012). The stimulating effect of warming on *A. nodosum* productivity at the northern edge of occurrence may continue well into the future, suggesting more productive intertidal communities in the future sub-arctic. Given the role of *A. nodosum* as key foundation species, climate change is expected to increase food and habitat provision to intertidal communities.

## Electronic supplementary material

Below is the link to the electronic supplementary material.

Supplementary material 1 (PDF 62 kb)

## Footnotes

1
www.g-e-m.dk.
