# Supplementary material for: Climate change stimulates the growth of the intertidal macroalgae Ascophyllum nodosum near the northern distribution limit
Source: Ambio. 2017 Jan 23;46(Suppl 1):119–31. doi: 10.1007/s13280-016-0873-7 (PMC5258665; doi:10.1007/s13280-016-0873-7)
Supplement: Supplementary file 1 — Supplementary material 1 (PDF 62 kb) [file 13280_2016_873_MOESM1_ESM.pdf]

Ambio

Electronic Supplementary Material

This supplementary material has not been peer reviewed

Title: Climate change stimulates the growth of the intertidal macroalgae *Ascophyllum nodosum* near the northern distribution limit

Authors: Núria Marbà, Dorte Krause-Jensen, Birgit Olesen, Peter B. Christensen, Anissa Merzouk, Joao Rodrigues, Susse Wegeberg, Robert T. Wilce

Table S1 Regression equations of temporal trends, for the entire time series and for the period since 1990, of annual mean air temperature, annual ice free days and *Ascophyllum nodosum* annual tip elongation rate at the study sites. The equations are of the form Y = a + (b· X). The p-value and the coefficient of determination are provided. The time periods of the series analysed are indicated.

Y	X	site	b	SE_b	a	SE_a	p-value	R2	time period
mean annual air temperature (oC)	time (year)	Tromsø	0.00	0.00	-2.96	3.45	0.08	0.03	1921-2015
		Tromsø	0.01	0.01	-10.92	23.96	0.56	0.02	1990-2012
		Lofoten	0.01	0.00	-20.44	3.21	<.0001	0.26	1900-2015
		Lofoten	0.02	0.01	-33.14	24.79	0.19	0.08	1990-2012
		Aasiaat	0.03	0.01	-63.98	24.15	0.02	0.10	1953-2012
		Aasiaat	0.25	0.02	-505.86	42.63	<.0001	0.87	1990-2012
		Sisimiut	0.04	0.01	-84.12	24.02	0.00	0.18	1961-2012
		Sisimiut	0.23	0.02	-462.28	44.03	<.0001	0.84	1990-2012
		Nuuk	0.00	0.01	-11.03	15.87	0.54	0.01	1953-2012
		Nuuk	0.13	0.02	-256.08	42.40	<.0001	0.63	1990-2012
annual ice-free days (days yr-1)	time (year)	Tromsø	-	-	-	-	-	-	1990-2009
		Lofoten	-	-	-	-	-	-	1990-2009

		Qeqertarsuaq	9.12	1.54	-18055.37	3083.33	<.0001	0.66	1990-2009
		Sisimiut	8.15	0.90	-16023.95	1791.25	<.0001	0.82	1990-2009
		Nuuk	2.56	0.71	-4773.49	1422.16	0.00	0.42	1990-2009
annual tip elongation (cm yr-1)	time (year)	Tromsø	0.09	0.06	-175.79	118.84	0.18	0.28	2002-2009
		Lofoten	0.24	0.03	-482.06	54.26	<.0001	0.88	1997-2009
		Kronprinsens	0.09	0.02	-169.95	31.06	<.0001	0.67	1995-2011
		Kronprinsens	0.03	0.01	-54.78	11.82	0.00	0.56	1958-2011
		Qeqertarsuaq	0.00	0.04	-4.99	79.53	0.92	0.00	1997-2008
		Sisimiut	0.01	0.03	-22.86	52.28	0.63	0.04	2000-2008
		Kobbe Inner	0.00	0.04	-0.94	72.56	0.93	0.00	1997-2009
		Kobbe Mid	0.17	0.06	-343.55	119.77	0.01	0.44	1997-2009
annual tip elongation (cm yr-1)	mean annual air temperature (oC)	Tromsø	0.49	0.44	4.33	1.61	0.32	0.17	2002-2009
		Lofoten	2.02	0.50	4.12	0.30	0.00	0.60	1997-2009
		Kronprinsens	0.40	0.10	4.23	0.32	0.00	0.50	1995-2011
		Kronprinsens	0.28	0.07	3.88	0.27	0.00	0.44	1958-2011
		Qeqertarsuaq	0.44	0.18	4.28	0.55	0.04	0.41	1997-2008

		Sisimiut	-0.05	0.12	3.56	0.20	0.70	0.02	2000-2008
		Kobbe Inner	-0.55	0.23	5.21	0.15	0.04	0.34	1997-2009
		Kobbe Mid	0.83	0.57	7.35	0.38	0.18	0.16	1997-2009
annual tip elongation (cm yr-1)	annual ice-free days (days yr-1)	Tromsø	-	-	-	-	-	-	2002-2009
		Lofoten	-	-	-	-	-	-	1997-2009
		Kronprinsens	0.003	0.002	2.32	0.34	0.07	0.23	1995-2011
		Qeqertarsuaq	0.000	0.002	3.05	0.48	0.88	0.00	1997-2008
		Sisimiut	0.001	0.002	3.44	0.55	0.72	0.02	2000-2008
		Kobbe Inner	0.002	0.016	0.91	5.54	0.92	0.00	1997-2009
		Kobbe Mid	0.082	0.024	-21.84	8.57	0.01	0.51	1997-2009

Table S2 Coefficients (and standard error of the coefficients), significance (p-value) and coefficient of determination (R^2) of the models fit between annual air temperature, annual ice-free days or *Ascophyllum nodosum* growth and time (years, period 1990-2012) and latitude across all studied Arctic and subarctic populations. The model was $Y = a_1 + b_1 \cdot time + a_2 \cdot latitude + b_2 (time \cdot latitude)$.

Y	Term	coefficient	Std Error	p-value	R^2
mean annual air temperature (^oC)	intercept	-305.16	63.02	<.0001	0.13
	time	0.15	0.03	<.0001	
	latitude	0.07	0.09	0.45	
	(latitude-67.3567)*(year-2000.73)	0.00	0.01	0.80	
annual ice-free days (days yr^{-1})	intercept	-8822.68	1885.24	<.0001	0.52
	time	5.37	0.94	<.0001	
	latitude	-24.38	2.62	<.0001	
	(latitude-66.9587)*(year-1999.59)	1.03	0.45	0.024	
annual tip elongation (cm yr-1)	intercept	-213.87	67.30	0.002	0.47
	time	0.12	0.03	0.000	
	latitude	-0.45	0.06	<.0001	
	(latitude-67.289)*(year-2003.27)	0.02	0.02	0.24	
